# Aldehyde dehydrogenase in solid tumors and other diseases: Potential biomarkers and therapeutic targets

**DOI:** 10.1002/mco2.195

**Published:** 2023-01-16

**Authors:** Jie Xia, Siqin Li, Suling Liu, Lixing Zhang

**Affiliations:** ^1^ Fudan University Shanghai Cancer Center & Institutes of Biomedical Sciences, State Key Laboratory of Genetic Engineering, Cancer Institutes, Key Laboratory of Breast Cancer in Shanghai, The Shanghai Key Laboratory of Medical Epigenetics, Shanghai Key Laboratory of Radiation Oncology, The International Co‐laboratory of Medical Epigenetics and Metabolism, Ministry of Science and Technology, Shanghai Medical College Fudan University Shanghai China; ^2^ Jiangsu Key Lab of Cancer Biomarkers, Prevention and Treatment, Collaborative Innovation Center for Cancer Medicine Nanjing Medical University Nanjing China

**Keywords:** aldehyde dehydrogenase, biomarker, cancer stem cell, disease, solid tumor, therapeutic resistance

## Abstract

The family of aldehyde dehydrogenases (ALDHs) contains 19 isozymes and is involved in the oxidation of endogenous and exogenous aldehydes to carboxylic acids, which contributes to cellular and tissue homeostasis. ALDHs play essential parts in detoxification, biosynthesis, and antioxidants, which are of important value for cell proliferation, differentiation, and survival in normal body tissues. However, ALDHs are frequently dysregulated and associated with various diseases like Alzheimer's disease, Parkinson's disease, and especially solid tumors. Notably, the involvement of the ALDHs in tumor progression is responsible for the maintenance of the stem‐cell‐like phenotype, triggering rapid and aggressive clinical progressions. ALDHs have captured increasing attention as biomarkers for disease diagnosis and prognosis. Nevertheless, these require further longitudinal clinical studies in large populations for broad application. This review summarizes our current knowledge regarding ALDHs as potential biomarkers in tumors and several non‐tumor diseases, as well as recent advances in our understanding of the functions and underlying molecular mechanisms of ALDHs in disease development. Finally, we discuss the therapeutic potential of ALDHs in diseases, especially in tumor therapy with an emphasis on their clinical implications.

## Graphical abstract

The human aldehyde dehydrogenase (ALDH) superfamily comprises 19 isozymes and can be divided into 11 distinct families. ALDHs are a group of nicotinamide adenine dinucleotide phosphate (NADP+)‐dependent enzymes that could catalyze the reversible oxidation of endogenous and exogenous aldehydes to their corresponding carboxylic acids. However, ALDHs are frequently dysregulated and associated with various diseases like Alzheimer's disease, Parkinson's disease, and especially solid tumors. Different ALDH isoenzymes are believed to be potential biomarkers or therapeutic targets in different diseases.

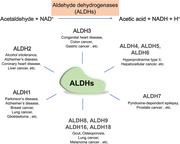

## 1 INTRODUCTION

The human aldehyde dehydrogenase (ALDH) superfamily comprises 19 isozymes and can be divided into 11 distinct families, which are respectively located in the cell cytoplasm, mitochondria, nucleus, and endoplasmic reticulum.^1^ Biologically, ALDHs are a group of nicotinamide adenine dinucleotide phosphate (NADP^+^)‐dependent enzymes that could catalyze the reversible oxidation of endogenous and exogenous aldehydes to their corresponding carboxylic acids,^2^ which protects living organisms against oxidative stress. ALDHs also take a role in scavenging reactive oxygen species (ROS) from aldehydes accumulation, thereby reducing oxidative stress in cells especially in stem cells.^3^, ^4^ Another vital function of ALDHs is catalyzing retinoic acid (RA) metabolism, which is crucial for normal embryonic development and epithelial differentiation.^5^

Throughout the past decades, pioneering studies support that the aberrant expression of ALDHs is related to several human non‐tumor diseases, for example, Alzheimer's disease (AD)^6^ and alcohol intolerance,^7^ which are caused by the loss of activity or deficiency of enzyme expression and accumulation of toxic aldehydes. Another ALDH‐related disorder is caused by ALDH gene mutations, especially single‐nucleotide polymorphisms,^8^ contributing to enzyme inactivation, cellular dysfunction, interruption of normal metabolic pathways, and susceptibility to human diseases, such as Sjögren–Larsson syndrome (SLS) and hyperprolinemia type II (HPII). Polymorphisms within these genes may differentially affect the risk of disease development across the ethnic groups evaluated. These increase the possibility of ALDHs as surrogate markers for identifying disease pathogenesis and clinicopathological characteristics.

It is noteworthy that aberrant ALDH activity or expression has been detected in different solid tumors, including breast cancer,^9^ colorectal cancer (CRC),^10^ lung cancer,^11^ head‐and‐neck squamous cell carcinoma (HNSCC),^12^, ^13^ prostate cancer (PCa),^14^ pancreatic cancer,^15^ bladder cancer,^16^ and glioblastoma (GBM).^17^ High ALDH activity is enriched in stem‐ /progenitor‐like cells that recapitulate tumor heterogeneity. Cancer stem cells (CSCs) are rare cancer cells within a tumor with sustained self‐renewal and differentiation abilities. ALDHs overexpressed in CSCs have been confirmed to promote tumor growth, metastasis, therapeutic resistance, and immune escape.^18^, ^19^, ^20^ Therefore, ALDHs stand out as biomarkers for CSCs in several cancers. Clinically, ALDHs are also considered indicators of poor prognosis in solid cancers. A systematic review and retrospective analysis of 1557 patients with advanced or metastatic solid cancers shows that high ALDH1 expression is distinctly associated with poorer overall survival (OS), especially in breast cancer, HNSCC, cervical cancer (CC), and ovarian cancers.^21^ Therefore, clinical assessment of ALDHs during tumor progression improves our understanding of CSC evolution dynamics and paves the way for therapeutic applications. For another, targeting ALDH isoenzymes or ALDH‐related pathways hold promising therapeutic implications via suppressing cancer progression, particularly for eradicating the CSC populations.

Here we summarize the research on ALDHs‐related diseases, especially ALDHs as promising biomarkers of CSCs and powerful predictors of clinical prognosis in selected solid tumors that have been extensively studied or discussed. And we then highlight the current in our understanding of the molecular mechanisms of ALDHs in disease development, emphasizing preclinical/clinical approaches to target ALDHs in solid tumors.

## 2 FUNCTIONS OF ALDHS IN DISEASE

The ALDH family is involved in several biological processes essential for cell survival along with cell protection, such as the detoxification of toxic aldehydes, protection from oxidative stress, and regulation of RA metabolism. However, ALDHs are frequently dysfunctional and related to different diseases especially in solid tumors.

### 2.1 ALDHs in aldehydes detoxification

The detoxification role of ALDHs is critically important for homeostasis. Endogenous aldehydes are generated during amino acids, vitamins, alcohols, neurotransmitters metabolism, and lipid peroxidation (LPO),^22^ whereas exogenous aldehydes are derived from the metabolism of a wide range of environmental agents.^2^, ^23^ Specifically, aldehyde accumulation may lead to oxidative stress and DNA damage via ROS production and LPO. However, ALDHs activate cellular antioxidant and free radical scavenging systems to attenuate oxidative stress and reduce DNA damage.^3^ Singh et al. found that blood acetaldehyde levels and blood glucose levels are absolutely increased in global Aldh1b1 knockout mice, indicating that Aldh1b1 plays a pivotal role in the link between alcohol consumption and diabetes.^24^ Individuals who possess the ALDH2*2 mutation (rs671) genotype lose enzyme activity and are unable to metabolize acetaldehyde, thereby contributing to oxidative stress and alcohol‐induced flushing reaction. Furthermore, alcoholics with the ALDH2*2 allele have an increased risk of suffering esophageal cancer (EC), HNSCC, CRC, and late‐onset AD.^25^, ^26^

In tumors, evidence points to that ALDHs such as ALDH1A1, ALDH1A3, and ALDH3A1 are critical drivers of chemotherapeutic and radiotherapeutic resistance in many solid tumors by detoxifying cytotoxic drugs and mitigating oxidative stress.^23^, ^27^ For example, silencing of the ALDH1A1 gene in human breast cancer cells increases their sensitivity to paclitaxel by triggering the production of ROS, and similar results are obtained with other anticancer agents such as doxorubicin, sorafenib, and staurosporine.^28^ Another example is that high levels of ALDH1A1 lead to the acquisition of epithelial–mesenchymal transition (EMT) and CSC properties as well as erlotinib resistance through clearance of reactive chlorine species/ROS in lung cancers. Knockdown or pharmacological inhibition of ALDH1A1 overcomes erlotinib resistance in vitro and in vivo.^29^

### 2.2 ALDHs in RA signaling

**FIGURE 1:**
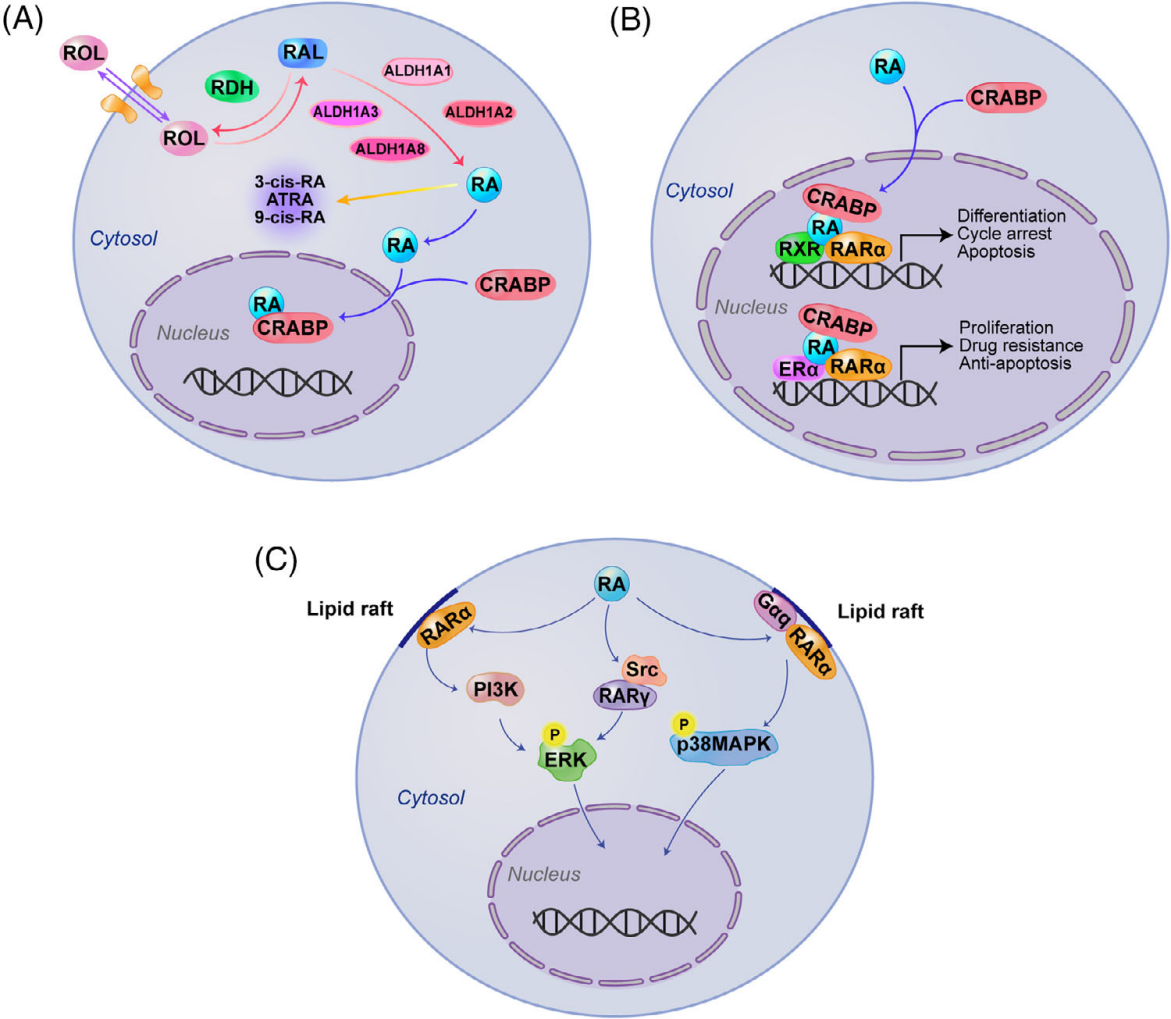
Aldehyde dehydrogenases (ALDHs) in retinoic acid (RA) signaling. (A) When retinols (ROLs) are absorbed by cells and reversibly oxidized to retinaldehydes (RALs) by retinol dehydrogenases (RDHs). Specific ALDH isozymes (ALDH1A1, ALDH1A2, ALDH1A3, and ALDH8A1) then catalyze the irreversibly nicotinamide adenine dinucleotide phosphate (NADP^+^)‐dependent oxidation of RALs to all‐*trans* RA (atRA), 9‐*cis*‐RA, and 13‐*cis*‐RA. (B) CRABPII transports RA to the nucleus where RA binds and activates heterodimers of nuclear RA receptors (RARs) or estrogen receptor α (ERα), respectively, on the RA response element (RARE), which can induce the transcriptional activity of target genes. (C) In response to RA, RARα translocates to lipid rafts in the cell membrane and interacts with Gαq proteins to activate the p38 mitogen‐activated kinase (p38MAPK) pathway. Besides, RARα and RARγ, via interaction with phosphoinositide 3‐kinase (PI3K) and non‐receptor tyrosine kinase (Src), respectively, can induce extracellular regulated–protein kinase (ERK) activation

RA signaling plays significant roles in embryonic stem cells,^30^ hematopoietic stem cells, cancer cells, and others.^31^ RA and its derivatives exert critical roles in regulating gene transcription involving cellular differentiation and proliferation^32^ as well as in maintaining epithelial homeostasis and immune response.^33^ Initially, dietary vitamin A or retinol (ROLs) are absorbed by cells and reversibly oxidized to retinaldehydes (RALs) by retinol dehydrogenases (Figure 1). Specific ALDH isozymes (ALDH1A1, ALDH1A2, ALDH1A3, and ALDH8A1) then catalyze the NADP^+^‐dependent oxidation of both all‐*trans*‐retinal and 9‐*cis*‐retinal to all‐*trans* RA (atRA), 9‐*cis*‐RA, and 13‐*cis*‐RA. This reaction is a tightly regulated, irreversible process.^34^ Newly synthesized RA can remain in the cell and bind to cellular RA–binding proteins (CRABPs). When bound to CRABPI, RA is targeted for degradation, whereas CRABPII translocates to the nucleus upon RA binding. RA could be translocated to the nucleus, where it binds and activates heterodimers of nuclear RA receptors (RARα, RARβ, and RARγ) and retinoic X receptors (RXRα, RXRβ, and RXRγ) on the RA response elements (RARE), which then induce the transcriptional activity of many genes involved in cell differentiation, cycle arrest, and apoptosis.^35^, ^36^, ^37^ Liu et al. showed that the concentration of serum RA determined by enzyme‐linked immunosorbent assay is significantly lower in type 2 diabetes mellitus (T2DM) patients than in normal glucose tolerance subjects.^38^, ^39^ To date, Han et al. confirmed that dysregulated serum RA levels can be used as biomarkers along with glucose for predicting future T2DM development in Korean subjects.^40^ In addition, studies have also revealed that circulating RA levels are negatively correlated with the development of coronary artery disease.^41^

In cancer, a well‐known example is that ALDH1A3/RA signaling activates the downstream factors homeobox transcription factor A1 (HOXA1) gene, which possesses a RARE sequence that is previously shown to be inducible by RA^35^ and is hypomethylated in breast cancer cell line MDA‐MB‐468 cells to be a tumor suppressor.^42^ In this respect, RAs have considerable clinical significance for the treatment of specific diseases.^43^

However, there are also reports demonstrating that RA can bind with other receptors, including estrogen receptor α or peroxisome proliferator‐activated receptors (PPAR) β/δ, which then activates oncogenes such as c‐MYC and cyclin D1 and thereby promotes tumor cell proliferation, drug resistance, and inhibition of apoptosis.^44^ A case in point is that RA treatment leads to parallel activation of RAR and PPAR β/δ to promote cancer cell survival in MMTV‐neu transgenic breast cancer mice.^45^, ^46^ Recently, a report also shows that atRA produced by ALDH1A1 transcriptionally activates functional RAREs in class III β‐tubulin (TUBB3) promoter, stimulating proliferation and sphere formation in patient‐derived bladder cancer cells.^47^ Generally, retinoid receptors are present in the nucleus. In some cases, however, they can be found in the cytoplasm, where they display nongenomic functions in a ligand‐dependent or independent manner, and as monomers or in complex with various factors.^48^, ^49^ Indeed, studies from several laboratories demonstrate that extranuclear effects of RA and RARs appear to be involved in different mechanisms and kinase cascades, including activation of p38 mitogen‐activated kinase (MAPK), extracellular regulated–protein kinase or p42/p44MAPK, phosphoinositide 3‐kinase (PI3K), and protein kinase B. As proof, in response to RA, RARα translocates to the lipid rafts in cell membrane where it interacts with Gα_q_ proteins and activates the MAPK pathway in breast tumor cells.^50^, ^51^ In addition, RA formed by ALDHs in cancer may affect cell‐autonomous pathways and trigger an immune cell fate switch. In sarcoma mouse models, Aldh1a1 or Aldh1a3 boosts atRA levels, which skews intra‐tumoral monocyte differentiation toward immunosuppressive macrophages. Pharmacological blockade of RA signaling in the tumor microenvironment increases immunostimulatory dendritic cells, enhances T‐cell‐dependent antitumor immunity, and synergizes with immune checkpoint inhibitors (ICIs).^33^

## 3 ALDHS AS DIAGNOSIS MARKERS IN NON‐TUMOR DISEASES

A sheer number of studies support that aberrant ALDH levels are related to several metabolic diseases and neurological abnormalities, which are mainly caused by dysregulated expression or enzyme inactivation.

### 3.1 ALDH1

*ALDH1A1*: Parkinson's disease (PD) is the second most common neurodegenerative disorder, characterized by the progressive loss of dopamine (DA) neurons in the substantia nigra pars compacta (SNpc).^52^ ALDH1A1 is an important molecular marker for the subpopulations of DA neurons in the SNpc that show differential susceptibility in PD‐related DA neurodegeneration, suggesting that the downregulation of ALDH1A1 expression may weaken its protective function against DA neurodegeneration in the ventral tier of SNpc. In support of this notion, a genetic deletion of Aldh1a1 exacerbates SNpc DA neuron loss in α‐synuclein transgenic mice, a murine model of PD‐related disease. However, the overexpression of ALDH1A1 is protective by preventing aldehyde risk factors, which indicates that ALDH1A1 is a potential therapeutic target in preventing PD pathogenesis.^53^ Besides in SNpc of PD patients, the mRNA expression of ALDH1A1 is also reduced in the peripheral blood of PD patients, which is classified as an optimal predictor for PD risk in a panel with four other genes.^54^, ^55^ However, it remains to be further determined by the large sample size. In contrast to PD, the role of ALDH1A1 in AD pathogenesis remains unclear. AD is the most frequent neurodegenerative disease.^56^ Although many studies have shown that ALDH1A1 is selectively expressed in the dopaminergic neurons, Fragoso et al. revealed that ALDH1A1 protein is also highly expressed in the human hippocampus, a region that is progressively degenerated in the brains of AD patients.^57^ Furthermore, by analyzing clinical human AD and age‐matched control brain tissues, Nikhil et al. showed that ALDH1A1 protein expression and activity are independently regulated and may not necessarily correlate with each other. During AD pathogenesis, ALDH1A1 activity is the highest at the initial stage, which can protect from accruing oxidative stress‐induced damage, but it declines significantly at the severe stage. In contrast, the ALDH1A1 protein level is significantly higher in severe AD tissues, but vastly compromised ALDH1A1 activity, which is presumably due to increased neurotoxicity.^58^ In addition, to gain insight into how ALDH1A1 neurons regulate behaviors of AD patients, by using a mutant Aldh1a1 mouse with AD, Li et al. found that Aldh1a1 neurons play an essential role in encoding a delay of gratification, and genetic deletion of Aldh1a1 causes impulsive behaviors in AD mice, which pinpoint a cellular point of entry to understand impulsive behaviors of AD patients.^59^ Recently, an ongoing clinical trial (NCT04878549) is being investigated to find a new approach by analyzing a 5‐gene transcription signature, including ALDH1A1 for the diagnosis of enteric fever induced by acute undifferentiated febrile infection, which indicates that ALDH1A1 may be a new marker for disease diagnosis.

*ALDH1A2*: ALDH1A2 is involved in the conversion of retinol into RA, which is an essential regulator of diaphragm/lung and cardiovascular development during human embryogenesis.^60^ In several animal models, downregulated RA results in cardiovascular, diaphragmatic, and associated pulmonary defects, which are consistent with the phenotype observed in patients.^61^ Furthermore, variants in the ALDH1A2 can affect downstream RA‐induced gene expression and cause lethal multiple congenital anomaly syndrome that is associated with pulmonary hypoplasia and respiratory failure.^62^ Lee et al. demonstrated that the synthesis of RA by ALDH1A2 marks mesoderm patterning and that specifies atrial cardiomyocytes from human pluripotent stem cells. This discovery provides new insights that can recreate aspects of cardiovascular disease in vitro and develop new therapeutic applications.^63^

*ALDH1B1*: ALDH1B1 is involved in ethanol detoxification, and modifications in this enzyme may contribute to alcohol‐related diseases. Some human ALDH1B1 polymorphism carriers are highly susceptible to alcohol‐associated diseases in Whites.^64^, ^65^ In agreement with these reports, Singh et al. generated global Aldh1b1 knockout mice that have an increase in blood acetaldehyde levels and blood glucose levels, indicating that Aldh1b1 has a potentially pivotal role in the link between alcohol consumption and diabetes.^24^

### 3.2 ALDH2

ALDH2 gene polymorphism is a potential risk marker for an array of cardiovascular anomalies and neurodegenerative diseases. The ALDH2*2 mutant (rs671) genotype, which is widely present in East Asians,^66^, ^67^ has significantly reduced the ALDH2 activity and induced the accumulation of aldehyde toxicity. A recent meta‐analysis of 5315 individuals by Chen et al. shows that the ALDH2∗2 polymorphism may be a potential risk factor for the development of AD,^68^ which is consistent with Kamino et al.^69^ and Ma et al.^70^ Another study provides evidence that ALDH2‐related signaling can be activated by melatonin and restore mitophagy and cardiomyocyte homeostasis in a mice model with AD. This is supported by the observation that the inhibition of ALDH2 eliminates melatonin‐mediated cardio‐protection.^71^ Notably, the group of Mochly‐Rosen also provides an important protection potential of ALDH2 against cardiac injuries,^72^ which is later confirmed by others.^73^, ^74^, ^75^ Upregulation of cellular ALDH2 activity can markedly reduce cytotoxic aldehydes and efficiently inhibit cardiac injury as well as significantly limit infarction size during cardiac ischemia in mouse models of myocardial infarction (MI) that can be reversed by the inhibition of ALDH2. Therefore, a clinical assessment of ALDH2 as a marker will be beneficial to the diagnosis of MI patients (NCT05360602). People with Asian flushing syndrome suffer alcohol intolerance and unfavorable cardiovascular conditions due to deficient activity of variant ALDH2∗2 allele.^76^ A clinical trial has been completed and evaluated whether the alcohol dehydrogenase inhibitor, fomepizole can treat symptoms associated with ALDH2 deficiency (NCT00661141).

Likewise, the risk of PD,^77^ hepatic steatosis,^78^ and coronary artery disease^79^ seems to be increased in individuals with the ALDH2*2 polymorphism, which is also confirmed by large‐scale meta‐analysis for risk assessment to develop essential hypertension.^80^

### 3.3 ALDH4

*ALDH4A1*: Interest in ALDH4A1 stems from the involvement of proline and hydroxyproline metabolism in many aspects of human health and disease. Pathogenic variants in the ALDH4A1 gene cause HPII, a metabolic disorder of the proline degradation pathway that can result in retardation and convulsion. Associated proteins of ALDH4A1 can potentially be used as pharmacological chaperones to stabilize the misfolded variants of ALDH4A1 in patients with HPII.^81^ In addition, patients with ALDH4A1 gene deficiency suffer from schizoaffective disorders and schizophrenia, and the potential molecular mechanism is also associated with abnormal proline metabolism.^82^ A recent study discovers that circulating ALDH4A1 is significantly elevated in the plasma of mice and humans with atherosclerosis, and the administration of ALDH4A1 antibody can protect against atherosclerosis progression. These results support that ALDH4A1 can be used as a putative disease biomarker and therapeutic target.^83^

### 3.4 Other ALDH isozymes

Studies using cell cultures and animal models have provided evidence that corneal ALDH3A1 and lens ALDH1A1 protect the eyes against cataract formation through non‐catalytic (light filtering) and catalytic (detoxification) mechanisms. Upon UVB exposure, ALDH3A1 gene knockout mice exhibit accelerated anterior lens subcapsular opacification, confirming a protective role of ALDH3A1 against cataract formation.^84^ Later, they show new evidence in vivo that ALDH3A1 is critical for maintaining corneal transparency for vision. Collectively, ALDH3A1 plays an important role in preventing cataract formation and holds great promise to be a therapeutic target.^85^ Mutations in the ALDH3A2 gene cause SLS, which will result in an abnormal accumulation of toxic fatty aldehydes in the brain and skin.^86^ Mutations in the ALDH5A1 gene cause succinic semialdehyde dehydrogenase (SSADH) deficiency, which is a genetic disease caused by the abnormal metabolism of γ‐aminobutyric acid (GABA).^87^ Mutations in the ALDH6A1 cause methylmalonic acidemia, which is a devastating metabolic disorder with a poor prognosis.^88^ Mutations in the ALDH7A1 cause pyridoxine‐responsive epilepsies, which is an inborn error of lysine catabolism that presents with refractory epilepsy in newborns.^89^ It also shows that variants in ALDH7A1 may impact patients’ risk of developing osteoporosis.^90^ Mutation in the ALDH16A1 gene can influence uric acid homeostasis and is associated with the pathogenesis of gout in humans and Mast syndrome.^91^, ^92^ Mutation in ALDH18A1 is related to cutis laxa syndromes as well as hyperammonemia due to amino acid abnormalities.^93^, ^94^ As discussed before and shown in Table 1, we summarize the findings on ALDHs as diagnostic markers in non‐tumor diseases.

**TABLE 1:** ALDHs as potential diagnostic markers in non‐tumor diseases

ALDH isoenzymes	Disease	Ref.
ALDH1A1	Cataract formation, Parkinson's disease, Alzheimer's disease	^52^, ^54^, ^55^, ^57^, ^58^, ^59^
ALDH1A2	Congenital heart disease, congenital anomaly syndrome	^61^, ^62^
ALDH1B1	Alcohol‐induced hypersensitivity, diabetes	^24^, ^64^, ^65^
ALDH2	Alcohol intolerance, hypertension, liver cirrhosis, Alzheimer's disease, Parkinson's disease, coronary heart disease, myocardial infarction	^66^, ^67^, ^68^, ^69^, ^70^, ^71^, ^72^, ^73^, ^74^, ^75^, ^76^, ^77^, ^78^, ^79^, ^80^
ALDH3A1	Cataract formation	^84^
ALDH3A2	Sjögren–Larsson syndrome	^86^
ALDH4A1	Hyperprolinemia type II, schizophrenia, atherosclerosis	^81^, ^82^
ALDH5A1	Succinic semialdehyde dehydrogenase deficiency	^87^
ALDH6A1	Methylmalonic academia	^88^
ALDH7A1	Pyridoxine‐dependent epilepsy, osteoporosis	^89^, ^90^
ALDH16A1	Gout; Mast syndrome	^91^, ^92^
ALDH18A1	Cutis laxa (CL) syndromes, hyperammonemia	^93^, ^94^

## 4 ALDHS AS CSC AND PROGNOSIS MARKERS IN TUMORS

Many studies have shown that CSCs are responsible for maintaining tumor heterogeneity, fueling tumor growth, and therapeutic resistance. For two decades, ALDHs have emerged as excellent biomarkers that can be used for the isolation and characterization of the CSCs population in solid tumors. The ALDEFLUOR assay^95^, ^96^ and immunostaining have proven useful in the identification and isolation of CSCs.^2^, ^97^ In the beginning, high ALDH activity enriched in CSCs is exclusively attributed to ALDH1A1, whereas in recent years this high activity has been associated with other isoforms,^98^ such as ALDH1A3^99^ in breast cancer, ALDH1B1^100^ in colon cancer, ALDH3A1^101^ in gastric cancer (GC), and ALDH7A1^102^ in PCa. In addition, ALDHs have long been regarded as potential prognostic markers in several cancer types, for example, ALDH3A1 in GBM,^17^ ALDH1L2 in melanoma,^103^ and ALDH18A1 in hepatocellular carcinoma (HCC).^104^ Next, we summarize the current knowledge regarding ALDHs as CSC markers or prognostic markers in selected solid cancers that have been extensively studied.

### 4.1 ALDH1

*ALDH1A1*: ALDH1A1 is a rate‐limiting enzyme involved in cellular RA synthesis as well as the oxidation of acetaldehydes and LPO‐derived aldehydes.^105^ Previously, ALDH1A1 is a major contributor to ALDH1 enzyme activity that could be detected by the ALDEFLOUR assay. The activity of ALDH1A1 is a reliable marker of CSCs in several types of solid tumors, including HNSCC,^12^, ^13^, ^106^ lung cancer,^11^ PCa,^14^ and bladder cancer.^16^ CSCs with high ALDH1A1 activity in these tumors are endowed with highly tumorigenic potential, enhanced capability of self‐renewal, and recapitulation of parental tumor heterogeneity. A well‐known example is that Ginestier et al. reported that ALDH1A1 is a general marker of both normal and cancer human mammary stem/progenitor cells. The marker can be inherited by progeny with the broadest self‐renewal capacity and lineage differentiation potential. These findings provide support for the “CSC hypothesis” and open new avenues for the study of normal breast organogenesis and breast carcinogenesis.^9^, ^107^ Later, Morimoto et al. investigated that ALDH1A1‐expressing breast cancers are shown to be more likely negative for ER and progesterone receptor (PR) expression, but positive for epidermal growth factor receptor 2 (HER2) and Ki‐67, correlating with more aggressive breast cancer subtypes.^108^ Charafe‐Jauffret et al. reported that ALDH1A1 contributes to aggressive behaviors by promoting tumor invasion in vitro and tumor metastasis in mouse xenografts. Moreover, ALDH1A1 expression is an independent predictive factor for early metastasis and decreased survival in inflammatory breast cancer.^109^ In two clinical trials to eliminate the activity of breast cancer stem cells (BCSCs) with bevacizumab (VEGF inhibitor) (NCT01190345) or reparixin (CXCR1/2 inhibitor) (NCT01861054), ALDH1A1 is one of the markers to measure the effect of drugs on BCSC activity. Consistent with breast cancer, ALDH1A1‐positive PCa and lung cancer cells display higher colony formation ability and sphere formation efficiency in vitro as well as greatly tumorigenic potential in vivo than ALDH1A1‐negative cells.^11^, ^14^ By analyzing clinicopathologic parameters, such as tumor stage, tumor grade, and lymph node metastasis (LNM), Kalantari et al. revealed that high ALDH1A1 expressions are significantly associated with PCa tumorigenesis and aggressive behaviors.^110^ Furthermore, ALDH1A1 is a marker of both normal stem cells and CSCs in colon tissues. When tracking the colon stem‐cell populations, ALDH1A1‐positive cells are a small subpopulation of cells (≤5%) localized at the bottom of normal crypts where stem cells reside but increase during the stepwise progression to colon cancer.^10^, ^111^

Accumulating data have confirmed that ALDH1A1 not only stands out as a CSC biomarker but is also associated with tumor metastasis and poor prognosis in different tumor types, such as breast cancer,^9^ PCa,^14^ HNSCC,^13^ bladder cancer,^16^ GBM,^112^ clear cell renal cell carcinoma (ccRCC),^113^ and GC.^114^ In breast tumors, consistent conclusions have shown that higher ALDH1A1 expression is associated with larger tumor size, higher histological grade, higher rates of LNM, higher expression of HER2, and lower expression of ER and PR.^115^, ^116^, ^117^, ^118^ Moreover, Kida et al. harbored the idea that ALDH1A1 expression is observably higher in triple‐negative breast cancer (TNBC) and HER2 subtypes compared to luminal subtype.^115^ Recently, ALDH1A1 drives nuclear factor‐κB and MAPK signaling, which promotes the expansion of myeloid‐derived suppressor cells (MDSCs) to thwart immune surveillance.^119^ Thus, shorter disease‐free survival (DFS), recurrence‐free survival (RFS), and/or OS are reported for patients with high levels of ALDH1A1 in breast tumors.^115^, ^120^ What's more, Zhou et al. are indicative of that ALDH1A1 is an independent prognostic factor in HNSCC patients, and the expression level of PDL‐1 may be involved in ALDH1A1‐mediated poor prognosis in patients.^121^ Additionally, ALDH1A1 contributes to the invasiveness of GBM and is an independent predictor of poor clinical outcomes in patients.^112^, ^122^ However, the clinical relevance of ALDH1A1 in CRCs remains controversial, although an association between the increased expression of ALDH1A1 and clinicopathological parameters, such as larger tumor size, higher histological grade, greater possibility of LNM has been observed across several studies.^105^, ^123^, ^124^, ^125^ Lugli et al. showed that 987 (76.7%) of the 1287 CRC tumors have negative cytoplasmic ALDH1A1 expression which indicates that ALDH1A1 expression is not associated with patient survival.^125^ However, Kahlert et al. found that the nuclear expression of ALDH1A1 is dramatically associated with shortened OS and DFS.^126^ Finally, Holah et al. concluded that the epithelial expression of ALDH1 may be associated with poor prognosis, whereas its stromal expression may be associated with good prognosis in CRC patients.^123^ By analyzing 4662 PCa cases and 3114 controls, Cao et al. found that genetic variants of ALDH1A1 in retinol metabolism pathway are closely related to tumor risk. The mutant (rs1330286) genotype in ALDH1A1 is associated with a low risk of PCa, whereas the mutant (rs4646653) genotype in ALDH1A3 is strongly related to a high risk of PCa, indicating that genetic variants in ALDH1A1 and ALDH1A3 may play different roles in the tumorigenesis.^127^

*ALDH1A2*: ALDH1A2 is involved in mediating synthesis of RA. Previous studies have demonstrated that ALDH1A2 serves as a candidate tumor suppressor. Low expression of ALDH1A2 is an unfavorable prognostic biomarker for survival in PCa,^128^ HNSCC,^129^ breast cancer,^130^, ^131^ ovarian cancer,^132^ and CC^133^ patients. Recently, Choi et al. delineated that ALDH1A2 is the most prominently downregulated gene among ALDH family members in ovarian cancer cells, based on microarray analysis. Besides, low ALDH1A2 expression is associated with shorter DFS and OS for ovarian cancer patients. Furthermore, functional assays confirm that the low expression of ALDH1A2 increases the potential of proliferation and invasion in ovarian cancer cells.^132^, ^134^ An immunohistochemistry (IHC) analysis of ALDH1A2 expression displays that its low expression is correlated with a shorter RFS in human PCa specimens, presumably due to its promoter hypermethylation.^128^ Notably, the ALDH1A2 gene is found to be hypermethylated via the DNMT1 or DNMT3B gene, consequently, promoting oncogenic activity.^132^ However, previous studies have found that ALDH1A2 and ALDH1B1 might be major contributors to the ALDH1 activity in non‐small cell lung cancers (NSCLCs) and a high expression of ALDH1A2 mRNA is significantly associated with poorer survival in patients,^135^ suggesting that ALDH1A2 may play different regulatory roles in different cancers.

*ALDH1A3*: ALDH1A3 is also one of important regulators in RA synthesis. It is universally acknowledged that ALDH1A3 takes an indispensable role in the generation and maintenance of CSCs in various solid cancers, that is, breast cancer,^99^ melanoma,^136^ ovarian cancer,^137^ PCa,^138^ GBM,^17^ lung cancer,^139^ pancreatic cancer,^140^ and CRC.^141^ ALDH1A3 is overexpressed in CSCs, always characterized by higher self‐renewal and tumor‐initiating capacity as well as drug resistance and worse prognosis in patients. In a report, including 58 human cancer cell lines, the ALDH1A3 mRNA expression is positively related to its ALDEFLOUR activity, implying that ALDH1A3 is one of the predominate ALDH isoenzymes to maintain the ALDH^bright^ populations.^141^ In PCa cells, ALDH1A3 is higher in primary PCa with luminal phenotype than in benign prostatic hyperplasia (BPH) tissues and normal tissues.^127^, ^138^, ^142^ Notably, several studies further investigated that high ALDH1A3 expression is correlated with worse progression‐free survival (PFS) for patients after prostatectomy^143^ and longer progression time to castration resistance for patients taking adjuvant hormonal therapy.^144^, ^145^ Besides, some groups have proposed that ALDH1A3 is an important breast CSC and prognosis marker.^42^, ^99^, ^146^ Marcato et al. published that the knockdown of ALDH1A3 robustly reduces ALDEFLOUR activity in breast cancer cells and tumors. More importantly, ALDH1A3 is positively correlated with breast cancer subtypes, tumor grade, and metastasis.^42^, ^99^ And in TNBCs and basal‐like subtypes, cancer cells have higher levels of ALDH1A3 expression, implying the prognostic value of ALDH1A3 in breast cancer patients.^147^ Interestingly, ALDH1A3 is highly expressed in mesenchymal glioma stem‐like cells (Mes‐GSCs) with a more aggressive phenotype, and the inhibition of ALDH1A3 attenuates the growth of Mes‐GSCs and sensitizes Mes‐GSCs to radiotherapy, suggesting that ALDH1A3 is a promising biomarker for Mes‐GSCs.^17^, ^148^, ^149^, ^150^ Mechanistically, forkhead box D1 protein triggers self‐renewal and tumorigenicity of Mes‐GSCs both in vitro and in vivo by regulating the transcriptional activity of ALDH1A3.^150^ Further studies indicate that ALDH1A1 or ALDH1A3 as markers of GSCs may be correlated with distinct molecular subtypes of high‐grade glioma (HGG) tumors, with ALDH1A3 being a marker of the mesenchymal subtype^17^, ^150^, ^151^ and ALDH1A1 being a marker of the classical subtype.^122^ Surprisingly, as reported by Luo et al., ALDH^bright^ melanoma‐initiating cells (MICs) highly express ALDH1A1 and ALDH1A3 isoenzymes, which have enhanced tumorigenic potential compared to ALDH‐negative cells^136^ and may be associated with distinct phenotypes of MICs. ALDH1A1 is predominantly expressed in human melanoma tumor samples, whereas ALDH1A3 is predominantly expressed in human melanoma cell lines.^152^

*ALDH1B1*: ALDH1B1 is a mitochondrial enzyme that can oxidize a broad range of substrates, including short‐ and medium‐chain aliphatic aldehydes, RALs and LPO‐derived products.^153^ A growing number of studies have revealed that the aberrant expression of ALDH1B1 has been observed in several human cancers such as CRC,^154^ pancreatic cancer,^15^ NSCLC,^135^ GC, and HCC,^155^ and it is involved in tumorigenesis and metastasis as well as clinical prognosis. By analyzing ALDH1B1 mRNA and protein levels, consistent conclusions suggest that ALDH1B1 levels distinguish CRC tissues from normal tissues.^156^, ^157^ Specifically, ALDH1B1 protein is 5.6‐fold higher than ALDH1A1 in CRC patients.^154^ In addition, Singh et al. showed that ALDH1B1 is a major contributor to ALDEFLOUR activity in highly tumorigenic colon cancer cells and can promote tumorigenesis by modulating the Wnt/β‐catenin, Notch and PI3K/Akt signaling pathways.^100^ In summary, ALDH1B1 is an excellent colon cancer biomarker. Afterward, ALDH1B1 has been shown to be a progenitor or stem‐cell marker during pancreas development and carcinogenesis.^15^, ^158^ ALDH1B1 is abundantly expressed in human pancreatic cancer and the high expression of ALDH1B1 contributes to cancer progression and dreadful prognosis.^105^, ^159^ Interestingly, ALDH1B1 expression is lower in HCC when compared with normal tissues, and lower ALDH1B1 expression is associated with an unfavorable prognosis in terms of OS and RFS,^155^ consistent with its roles in GC patients.^114^ Polymorphisms of ALDH1B1 can cause marked reductions in acetaldehyde metabolism ability and consequently result in flushing syndrome and ethanol avoidance. Studies reported that tobacco and/or alcohol consumption in carriers of the ALDH1B1 polymorphism increases the risk of oral squamous cell carcinoma (OSCC).^160^ A clinical trial (NCT04270201) to assess the relationship between ALDH1B1 polymorphism frequency and OSCC risk in the Brazilian population is underway. Further research using large prospective patient cohorts are warranted to determine the prognostic value of ALDH1B1 in certain cancers.

*ALDH1L1*: ALDH1L1, also known as 10‐formyltetrahydrofolate (10‐formyl‐THF) dehydrogenase, one of the folate‐metabolizing enzymes, catalyzes the oxidization of 10‐formyl‐THF to generate CO_2_ with concomitant NADPH production.^161^ Studies have established that ALDH1L1 is often strongly and universally downregulated or silenced in a multitude of human solid cancers, including HCC,^162^, ^163^, ^164^ lung adenocarcinoma (LUAD),^165^ ccRCC,^166^ neuroblastoma (NB),^167^ and breast cancer.^168^ Data indicate that the ALDH1L1 gene promoter is frequently hypermethylated in cancer, with low mRNA levels predicting poorer clinical outcomes.^165^, ^166^, ^168^, ^169^, ^170^ For example, the ALDH1L1 gene is hypermethylated in breast tumors after acquisition of chemoresistance^171^ and may be important for tumor cell survival in response to metabolic stress.^172^ Overall, ALDH1L1 could be a candidate tumor suppressor for aggressive cancers. However, the prognostic role of ALDH1L1 may be cancer‐type‐specific. Li et al. reported that ALDH1A3 and ALDH1L1 are potentially major contributors to ALDH1 activities in GC, and high mRNA expressions of ALDH1A3 and ALDH1L1 predict poorer OS in GC patients.^114^ Genetic variation analyses show that the ALDH1L1 variant rs2276724 genotype is of prognosis value in hepatitis B virus–related HCC patients and the carriers of rs2276724 are more probably with a favorable prognosis.^173^ Further well‐designed, comprehensive, and large sample size studies are therefore needed to confirm these results.

*ALDH1L2*: ALDH1L2 is an important mitochondrial paralog of ALDH1L1 and the product of a separate gene.^174^ Although both ALDH1L1 and ALDH1L2 are involved in folate metabolism, their compartmentalization results in distinct effects on overall cellular metabolism, regulating either folate pools and purine biosynthesis (cytosolic ALDH1L1) or NADPH production and oxidative stress (mitochondrial ALDH1L2). ALDH1L2 is highly expressed and presents as an independent prognostic factor for OS and RFS in melanoma, pancreatic ductal adenocarcinoma (PDAC), CRC, and GBM.^103^, ^175^ Several studies have shown that the depletion of ALDH1L2 markedly reduces NADPH/NADP^+^ and glutathione/oxidized glutathione redox couple ratios, reduces circulating tumor cells (CTCs) in the blood, and inhibits distant metastasis.^103^, ^175^, ^176^ On the other hand, evidence suggests that ALDH1L2 combats oxidative stress by increasing total cellular NADPH, which is responsible for GSC maintenance and growth.^177^

### 4.2 ALDH2

ALDH2 is known for its role in the metabolism of ethanol‐derived acetaldehydes.^178^, ^179^ Notably, ALDH2 dysfunction has been widely reported to be associated with tumorigenesis and tumor progression, which is often considered a feasible prognostic marker in different solid tumor types. Low ALDH2 expression is always responsible for poorer survival and more aggressive behaviors in liver cancer patients. ALDH2 can participate in inhibiting HCC cells metastasis via the adenosine 5′‐monophosphate‐activated protein kinase pathway,^180^ promoting HCC cell's autophagy and repressing its immune escape by blocking the ROS/Nrf2 axis.^181^ Remarkably, it is indicating that the polymorphisms of ALDH2 might have an important influence on liver cancer. The ALDH2*2 mutant (rs671) genotype is a shared missense mutation in the ALDH2 gene with severely reduced activity of the ALDH2 enzyme,^182^ which has been found to increase protein turnover and promote hepatocarcinogenesis in vivo.^183^ More recently, the polymorphism of ALDH2 gene is reported to be a risk factor with a prognostic value for alcohol‐related cancers, including HNSCC, EC, HCC, CRC, GC, and breast cancer.^184^ Furthermore, a lower expression of ALDH2 in these tumors is associated with poorer OS and PFS.

However, there are also several studies emphasize that ALDH2 overexpression or high activity is associated with cancer progression and multidrug resistance.^185^ Chen et al. reported that ALDH2 promotes the expression of cancer stem genes (e.g., Nanog, Oct4, and Sox2), leading to the proliferation, migration, and invasion of CD133^+^CD24^+^ Huh‐7 liver CSCs.^186^ Wang et al. found that the expression of ALDH2 is also upregulated in NSCLC cells, which is associated with cancer cell stemness property and paclitaxel resistance.^187^ More implementation is needed to outline a possible role for ALDH2 in cancer.

### 4.3 ALDH3

*ALDH3A1*: ALDH3A1 participates in oxidation of alcohol‐derived acetaldehydes and in the metabolism of corticosteroids, biogenic amines, neurotransmitters, and LPO.^188^ In recent years, ALDH3A1 has been shown to be upregulated in several cancer types, for example, GC,^101^ lung cancer,^189^, ^190^ HCC,^191^ colon cancer,^192^ PCa^193^ and can be used as a biomarker to predict poor clinical outcomes. It has been demonstrated by Wu et al. that ALDH3A1 is upregulated in GC stem cells, which contributes to gastric carcinogenesis, and the high expression of ALDH3A1 protein is associated with a poorly differentiated state in GC tissues.^101^ In addition, the IHC analysis of GC patient specimens demonstrates that ALDH3A1 expression is closely correlated to clinical features, including cancer dysplasia and grade, LNM, and cancer stage.^101^, ^194^ Collectively, ALDH3A1 is a known CSC and prognosis marker for GC. More recently, publicly available data from The Cancer Genome Atlas (TCGA) find that ALDH3A1 is highly expressed in LUAD patients with metastasis, linked to the characteristics of CSCs and EMT. Besides, high ALDH3A1 expression predicts a poor prognosis in LUAD.^195^ Several groups have provided supportive evidence that ALDH1A1 and ALDH3A1 are expressed at significantly high levels in NSCLC that indicates poorer OS.^189^, ^196^, ^197^ Correspondingly, findings indicate that ALDH3A1 boosts tumor initiation and progression in PCa. ALDH3A1 shows high expression levels under sphere‐forming conditions in vitro by DU145 prostate CSCs.^193^ As the association of ALDH1A1 and ALDH7A1 with PCa progression has been definitively demonstrated,^102^, ^138^ it is proposed that high levels of ALDH3A1, ALDH1A1, and ALDH7A1 are involved in prostate tumorigenesis, though the detailed contribution of them to PCa requires further investigation.

*ALDH3A2*: ALDH3A2 catalyzes the oxidation of fatty aldehydes, and mutation in the ALDH3A2 gene results in SLS.^86^ Analysis of clinicopathological features reveals an association of reduced ALDH3A2 with advanced and poorly differentiated cancers. For instance, gene set enrichment analysis and IHC results indicate that high ALDH3A2 expression is associated with superior OS of GC patients. Moreover, ALDH3A2 is negatively correlated with immune checkpoints, including cytotoxic T‐lymphocyte‐associated antigen 4, programmed cell death protein 1 in immune cells, and its ligand PD‐L2 in GC cells.^198^ More recently, Antonowicz et al. supported that the loss of ALDH3A2 expression is linked to malignant transformation and progression and is associated with poorer survival in esophageal adenocarcinoma.^199^ Surprisingly, ALDH3A2 represents a very intriguing isoform as its expression is downregulated but increased in response to various treatment trials in PCa.^200^ Altogether, these findings potentially hint at a tumor suppressor role of ALDH3A2.

*ALDH3B1*: ALDH3B1 is generally thought to detoxify aldehydes from alcohol metabolism and LPO.^201^ ALDH3B1 is significantly engineered to be overexpressed in lung cancer^202^ and is positively correlated with patient tumor size and histological grade, indicating that ALDH3B1 is an independent prognostic biomarker of lung cancer patients.^203^ ALDH3B1 and ALDH16A1 are preferentially overexpressed in HGG and can promote the proliferation and migration of glioma cells by regulating cell cycle and EMT processes. Furthermore, high expressions of ALDH3B1 and ALDH16A1 are positively associated with worse treatment response and shorter OS in GBM patients.^204^

*ALDH3B2*: ALDH3B2 may play a major role in the detoxification of aldehydes generated by alcohol metabolism and LPO.^205^ Previous data suggest that ALDH3B2 gene polymorphisms are associated with susceptibility to CRC and ESCC.^206^ Furthermore, ALDH3B2 protein expression is significantly higher in the RCC tissues compared to the normal renal tissues, and ALDH3B2 independently predicts worse OS in patients.^207^ More recently, Wang et al. demonstrated that high expressions of ALDH3B2 and integrin beta 1 are strong inferior prognostic biomarkers in cholangiocarcinoma (CCA) patients.^208^

### 4.4 ALDH4

*ALDH4A1*: ALDH4A1, also known as delta‐1‐pyrroline‐5‐carboxylate (P5C) dehydrogenase, is involved in the metabolism of the amino acid arginine, proline and l‐valine.^34^ ALDH4A1 can serve as a potential disease indicator, in which circulating ALDH4A1 is increased during atherosclerosis in mice and humans and anti‐ALDH4A1 antibodies can protect against atherosclerosis progression.^83^ However, the precise role of ALDH4A1 in cancer progression and patient prognosis remains unclear. Previously, ALDH4A1 expression is transcriptionally activated by p53 to prevent the proline‐dependent ROS production, which confers a survival advantage to U373MG GBM cells and H1299 NSCLCs.^209^ Additionally, polymorphisms of ALDH4A1, ALDH18A1, ALDH3B2, ALDH1L2, ALDH1A2, and ALDH2*2 are significantly associated with an elevated nasopharyngeal carcinoma risk.^210^

### 4.5 ALDH5

*ALDH5A1*: ALDH5A1, which encodes for SSADH, is an enzyme involved in mitochondrial glutamate metabolism.^211^ ALDH5A1 converts succinic semialdehyde into succinate, fueling the TCA cycle and thereby limiting the γ‐hydroxybutyrate (GHB) production.^212^ It is reported that high ALDH5A1 expression is associated with stem‐cell‐like properties and aggressive behaviors within the context of GBM. On the contrary, the inhibition of ALDH5A1 leads to the accumulation of GHB that induces GBM stem‐like cell differentiation and reduces the aggressive phenotype.^213^ RNA sequencing analysis of human breast ductal carcinoma in situ (DCIS) cells demonstrates that ALDH5A1 is overexpressed at both the mRNA and protein levels. Two independent drugs, disulfiram (DSF)^214^ and valproic acid,^215^ inhibit ALDH5A1 activity to reduce proliferation of DCIS.^216^ More recently, the analysis results from the TCGA database show that ALDH5A1 is an excellent prognostic factor in patients with primary papillary thyroid cancer (PTC), manifesting that the high expression of ALDH5A1 predicts a worse prognosis. Mechanically, ALDH5A1 can enhance the migration and invasion ability of thyroid cancer cells through EMT transition, suggesting that ALDH5A1 may emerge as a new target for PTC therapy.^217^ Conversely, mRNA and protein expressions of ALDH5A1 are lower in HCC and ovarian cancer that predicts poorer OS in patients.^218^ A recent study by Kong et al. discovers that ALDH5A1 is upregulated upon the knockdown of a prostate‐specific antigen in PCa cells, accompanied by reduced tumorigenesis and metastasis in vitro and in vivo.^219^ Hence, ALDH5A1 may act as an oncogene or tumor suppressor in various human cancers and is strongly associated with the clinical outcomes.

### 4.6 ALDH6

*ALDH6A1*: ALDH6A1 is a mitochondrial methylmalonate semialdehyde dehydrogenase that plays a vital role in the valine and pyrimidine catabolic pathways. ALDH6A1 catalyzes the irreversible oxidative decarboxylation of methylmalonate semialdehyde to acetyl‐CoA and propionyl‐CoA.^220^ Extensive clinical analyses have shown that the decreased expression of ALDH6A1 is correlated to tumorigenesis and inferior outcomes in different kinds of cancers, which may serve as a potential diagnostic and prognostic biomarker. ALDH6A1 expression is suppressed in HCC, which is accompanied by an elevation of NO levels and a reduction of ROS levels that may support abnormal HCC cell growth.^221^ Expressions of 4‐aminobutyrate aminotransferase and ALDH6A1 are significantly reduced in ccRCC patients, which are correlated with poorer survival. Overexpression of ALDH6A1 reduces cell growth and migration and impairs tumor metabolism of ccRCC cells.^222^ In addition, ALDH6A1 is significantly reduced in metastatic PCa compared with normal and primary PCa, an indication of that ALDH6A1 may be a predictive biomarker for metastatic PCa.^223^ Consistent with these reports, ALDH6A1 also functions as a tumor suppressor in bladder cancer. ALDH6A1 is remarkably downregulated in bladder cancer tissues and cell lines. Low expression of ALDH6A1 is positively associated with advanced cancer subtype and cisplatin resistance, hinting at a poorer outcome in bladder cancer patients.^224^

### 4.7 ALDH7

*ALDH7A1*: ALDH7A1 could function in the protection of cells from oxidative stress by metabolizing a number of LPO‐derived aldehydes and function in lysine catabolism.^225^ ALDH1A1 and ALDH7A1 are previously found to contribute to ALDEFLOUR activity in prostate CSCs.^102^ Besides, a high expression of ALDH7A1 is suggested to predict disease progression and metastasis. van den Hoogen et al. delineated that the mRNA expression levels of several ALDH isoforms, including ALDH7A1, are evaluated in PCa clinical specimens and cell lines. Notably, strong ALDH7A1 expression triggers PCa bone metastasis.^102^, ^226^ Indeed, the knockdown of ALDH7A1 results in a significant reduction of ALDH^bright^ populations and significantly inhibits clonogenicity and cell invasion of human PCa cells in vitro.^102^ Subsequent studies demonstrate that ALDH1A3, ALDH7A1, and ALDH18A1 isoforms are more robustly expressed in PCa than in BPH and normal samples.^138^ Similarly, low ALDH7A1 expression is associated with a lower incidence of cancer recurrence and superior RFS and OS in surgically resected NSCLC patients.^227^ Recently, significantly reduced methylation levels of ALDH7A1 gene are observed in lung SCC with idiopathic pulmonary fibrosis compared with patients without fibrosis, which predicts poor outcome.^228^ Taken together, ALDH7A1 has the potential to be a clinically useful biomarker in cancers.

### 4.8 ALDH8

*ALDH8A1*: ALDH8A1 is critical for catalyzing RALs to the corresponding RA. Bioinformatics analysis by Chen et al. reveals that ALDH8A1 has higher levels of promoter DNA methylation in CCA.^229^ And in liver cancer, an eight‐gene signature expression, including ALDH8A1, is significantly downregulated in cancer tissues, and low expression can be regarded as an inferior prognostic biomarker.^230^

### 4.9 ALDH9

*ALDH9A1*: ALDH9A1 is an enzyme that encodes γ‐trimethylaminobutyraldehyde dehydrogenase that catalyzes γ‐aminobutyraldehyde to GABA.^231^ However, knowledge about the aberrant ALDH9A1 expression on cancer development remains elusive. The abundance of ALDH1A1, ALDH4A1, ALDH6A1, ALDH7A1, and ALDH9A1, which are known to be involved in xenobiotic metabolism signaling, is elevated in RCC.^232^ In another comparative study, Stevenson et al. showed that ALDH1A1, ALDH2, and ALDH9A1 proteins are present in LNM specimens from breast cancer, PDAC and PCa, indicating a potential common role of these proteins in the development of LNM.^233^

### 4.10 ALDH16

*ALDH16A1*: The function of ALDH16A1 reported in cancer is limited. Analysis by Wang et al. manifested that ALDH16A1 is considered a prognostic biomarker in gliomas. They show that ALDH2, ALDH5A1, ALDH6A1, ALDH1L2, ALDH1B1, ALDH18A1, ALDH1A1, ALDH8A1, and ALDH1B1 expressions are higher in isocitrate dehydrogenase (IDH) mutant gliomas and demonstrated to be favorable factors for patients with low‐risk scores, whereas ALDH16A1, ALDH3B1, ALDH3A1, ALDH7A1, ALDH1A3, ALDH1L1, and ALDH1A2 expressions are increased in IDH wildtype and are associated with a worse prognosis risk.^204^

### 4.11 ALDH18

*ALDH18A1*: ALDH18A1 is a mitochondrial enzyme that catalyzes the conversion of l‐glutamate to P5C, an important intermediate step in proline metabolism.^234^ ALDH18A1 overexpression locks melanoma cells into a more proliferative or tumorigenic state via proline biosynthesis.^235^ ALDH18A1 knockdown decreases intracellular proline levels and impairs melanoma cell viability and tumor progression. Thus, the proline synthesis pathway can be therapeutically targeted by inhibiting ALDH18A1 in melanoma.^236^ ALDH9A1 and ALDH18A1 are dramatically upregulated in CTCs of PCa compared to primary tumors.^237^ Moreover, the high expression of ALDH18A1 predicts a poor clinical outcome in HCC,^104^ NSCLC,^238^, ^239^ and breast cancer.^240^ ALDH18A1 is overexpressed in highly proliferative luminal B compared to low proliferative luminal A breast cancer subtypes.^240^ A recent study reveals that ALDH18A1 forms a positive feedback loop with MYCN and is involved in the regulation of the proliferation, self‐renewal and tumorigenicity of NB cells. Besides, the high expression of ALDH18A1 is linked to poorer OS and event‐free survival in the Kaplan–Meier plot. Targeting ALDH18A1 with a specific inhibitor, YG1702, dictates MYCN expression suppression and attenuates NB growth in preclinical xenograft models.^241^

Based on the previous discussion, we further summarize the findings about characteristics of ALDHs in tumors in Table 2. However, the precise roles of ALDH isoenzymes in cancer cells with a stem‐cell‐like phenotype or clinical prognostic significance require further elucidation as they may greatly vary by cancer types and tissues of origin.

**TABLE 2:** Characteristics of aldehyde dehydrogenases (ALDHs) family in tumors

	ALDH isoenzymes	Chromosomal location	Subcellular localization	Functional activity	CSC marker	Prognostic value	Clinical trials	Ref.
ALDH families								
ALDH1	ALDH1A1	9q21.13	Cytosol	Oxidation of RALs to RAs; oxidation of acetaldehydes and LPO‐derived aldehydes	Breast cancer, HNSCC, GBM, lung cancer, ovarian cancer, GC, PCa, bladder cancer, CRC, etc.	High expression predicts unfavorable prognosis in patients with breast cancer, HNSCC, GBM, EC, GC, lung cancer, PCa, bladder cancer, and ccRCC, etc.	NCT01190345 (Phase II/completed) NCT01861054 (Phase II/terminated) NCT04878549 (not applicable/recruiting)	^9^, ^10^, ^11^, ^13^, ^14^, ^16^, ^110^, ^112^, ^113^, ^114^
	ALDH1A2	15q21.3	Cytosol	Oxidation of RALs to RAs		High expression predicts favorable prognosis in patients with PCa, HNSCC, breast cancer, ovarian cancer, CC, etc. High expression predicts unfavorable prognosis in patients with NSCLC, etc.		^128^, ^129^, ^130^, ^132^, ^133^, ^135^
	ALDH1A3	15q26.3	Cytosol nucleus	Oxidation of RALs to RAs	Pancreatic cancer, PCa, melanomas, GBM, ovarian cancer, lung cancer, CRC, breast cancer, etc.	High expression predicts unfavorable prognosis in patients with pancreatic cancer, GBM, PCa, ovarian cancer, lung cancer, CRC, melanoma, breast cancer, etc.		^17^, ^99^, ^136^, ^137^, ^138^, ^139^, ^140^, ^141^
**ALDH subfamilies**								
ALDH1	ALDH1B1	9p13.1	Mitochondria nucleus cytosol	Oxidation of acetaldehydes and LPO‐derived aldehydes	CRC, pancreatic cancer, etc.	High expression predicts unfavorable prognosis in patients with CRC, pancreatic adenocarcinoma, NSCLC, etc. High expression predicts favorable prognosis in patients with HCC, GC, etc.	NCT04270201 (not applicable/not yet recruiting)	^15^, ^100^, ^114^, ^135^, ^154^, ^155^
	ALDH1L1	3q21.3	Cytosol	Conversion of 10‐formyl‐THF in tetrahydrofolate		High expression predicts favorable prognosis in patients with LUAD, HCC, ccRCC, NB, breast cancer, etc. High expression predicts unfavorable prognosis in patients with GC, etc.		^114^, ^162^, ^165^, ^166^, ^167^, ^168^
	ALDH1L2	12q23.3	Mitochondria	Conversion of 10‐formyl‐THF in tetrahydrofolate		High expression predicts unfavorable prognosis in melanoma, PDAC, CRC, GC, GBM, etc.		^103^, ^175^, ^176^, ^177^
**ALDH subfamilies**								
ALDH2	ALDH2	12q24.12	Mitochondria	Oxidation of alcohol and LPO‐derived acetaldehydes		High expression predicts favorable prognosis in patients with liver cancer, lung cancer, breast cancer, EC, CRC, GC, HNSCC, etc.	NCT00661141 (Phase II/Completed) NCT05360602 (Not applicable/Not yet recruiting)	^180^, ^184^
ALDH3	ALDH3A1	17p11.2	Plasma membrane cytosol	Oxidation of alcohol‐derived acetaldehydes and in the metabolism of corticosteroids, biogenic amines, neurotransmitters, and LPO	GC, lung cancer, HCC, colon cancer, PCa, etc.	High expression predicts unfavorable prognosis in patients with GC, LUAD, NSCLC, HCC, PCa, colon cancer, etc.		^101^, ^189^, ^190^, ^191^, ^192^, ^193^
	ALDH3A2	17p11.2	Endoplasmic reticulum Peroxisomes	Oxidation of long‐chain aliphatic‐aldehydes into fatty acids		High expression predicts favorable prognosis in patients with GC, EAC, PCa, etc.		^198^, ^199^, ^200^
**ALDH subfamilies**								
ALDH3	ALDH3B1	11q13.2	Plasma membrane cytosol	Detoxification of aldehydes from alcohol metabolism and LPO		High expression predicts unfavorable prognosis in patients with lung cancer, GBM, etc.		^202^, ^203^, ^204^
	ALDH3B2	11q13.2	Lipid droplet	Detoxification of aldehydes from alcohol metabolism and LPO		High expression predicts unfavorable prognosis in patients with RCC, CCA, etc.		^207^, ^208^
ALDH4	ALDH4A1	1p36.13	Mitochondria	Metabolism of the amino acid arginine, proline, and l‐valine				
**ALDH subfamilies**								
ALDH5	ALDH5A1	6p22.3	Mitochondria	Conversion of succinic semialdehyde in succinate		High expression predicts unfavorable prognosis in patients with GBM, DCIS, PTC, etc. High expression predicts favorable prognosis in patients with HCC, ovarian cancer, etc.		^213^, ^216^, ^217^, ^218^, ^219^
ALDH6	ALDH6A1	14q24.3	Mitochondria	Oxidation of methylmalonate semialdehyde to acetyl‐CoA and propionyl‐CoA; Involvement in valine, and pyrimidine metabolism		High expression predicts favorable prognosis in patients with HCC, ccRCC, PCa, bladder cancer, etc.		^221^, ^222^, ^223^, ^224^
ALDH7	ALDH7A1	5q23.2	Nucleus Cytosol mitochondria		PCa	High expression predicts unfavorable prognosis in patients with PCa, NSCLC, lung SCC, etc.		^102^, ^226^, ^227^, ^228^
**ALDH subfamilies**								
ALDH8	ALDH8A1	6q23.3	Cytosol	Oxidation of RALs to RA		High expression predicts favorable prognosis in patients with liver cancer		^230^
ALDH9	ALDH9A1	1q24.1	Cytosol	Oxidation of γ‐aminobutyraldehyde to GABA				
ALDH16	ALDH16A1	19q13.33	Plasma membrane cytosol	Not fully discovered		High expression predicts unfavorable prognosis in patients with gliomas		^204^
ALDH18	ALDH18A1	10q24.1	Mitochondria	Conversion of l‐glutamate to P5C		High expression predicts unfavorable prognosis in patients with HCC, NSCLC, breast cancer, melanoma cancer, PCa, NB, etc.		^104^, ^236^, ^237^, ^238^, ^240^, ^241^

Abbreviations: 10‐formyl‐THF, 10‐formyltetrahydrofolate; CC, cervical cancer; CCA, cholangiocarcinoma; ccRCC, clear cell renal cell carcinoma; CRC, colorectal cancer; CSC, cancer stem cell; DCIS, breast ductal carcinoma in situ; EAC, esophageal adenocarcinoma; EC, esophageal cancer; GABA, γ‐aminobutyric acid; GBM, glioblastoma; GC, gastric cancer; HCC, hepatocellular carcinoma; HNSCC, head‐and‐neck squamous cell carcinoma; LPO, lipid peroxidation; LUAD, lung adenocarcinoma; NB, neuroblastoma; NSCLC, non‐small cell lung cancers; P5C, pyrroline‐5‐carboxylate; PCa, prostate cancer; PDAC, pancreatic ductal adenocarcinoma; PTC, papillary thyroid cancer; RA, retinoic acid; RAL, retinaldehyde.

## 5 THERAPEUTIC POTENTIAL OF TARGETING ALDHS

As mentioned before, ALDHs are widely accepted as functional biomarkers and regulators of stemness phenotypes in many diseases. Here, we further review the studies concerning the therapeutic potential of targeting ALDHs in diseases, especially solid tumors.

### 5.1 Targeting ALDHs with ALDH inhibitors

ALDH inhibitors can be classified into multi‐ALDH isoform inhibitors and isoform‐specific inhibitors which primarily inhibit one isoform.^178^, ^242^ The multi‐ALDH isoform inhibitors include DSF, diethylaminobenzaldehyde (DEAB), 4‐dimethylamino‐4‐methyl‐pent‐2‐ynthioic acid‐*S*‐methylester (DIMATE), citral, and aldehyde dehydrogenase inhibitors 1–4 and 6, dyclonine (Table 3).

**TABLE 3:** Multi‐ALDH (aldehyde dehydrogenase) isoform inhibitors

Inhibitor	Target	Cellular activity	Animal/clinical studies	Ref.
DSF	Irreversible ALDH inhibitor	IC50: 0.15 μM for ALDH1, and 1.45 μM for ALDH2	DSF/Cu can inhibit ALDH‐positive NSCLC tumors. The combination of DSF and gemcitabine efficiently inhibits breast tumors; the combination of TMZ and DSF/Cu in a Phase II clinical trial (NCT03034135) rescue ALDH1A3‐mediated TMZ resistance in GBM patients	^243^, ^244^, ^245^, ^246^, ^247^
DEAB	A reversible substrate for ALDH1A1 and ALDH3A1, and displays competitive inhibition of ALDH1A1, ALDH1A3, ALDH1B1, and ALDH5A1	IC50: 57 nM for ALDH1A1, 1.2 μM for ALDH1A2, 3 μM for ALDH1A3, 1.2 μM for ALDH1B1, 0.16 μM for ALDH2, and 13 μM for ALDH5A1	Inhibition of tumor growth and pulmonary metastasis in breast cancer tumors	^95^, ^248^
DIMATE	ALDH1 and ALDH3 subfamilies	IC50: 5 μM for ALDH1A1 and ALDH3A1	DIMATE is effective at a dose of 14 mg/kg daily given via intraperitoneal injection in PCa tumors	^249^, ^250^
Citral	Reversible noncompetitive ALDH inhibition	Potent inhibitory activity against ALDH1A1, ALDH1A3, and ALDH2	Nanoparticle encapsulated citral reduces the tumor growth of ALDH1A3‐overexpressing MDA‐MB‐231 xenografts	^250^, ^251^
Aldi‐1–4	Covalent ALDH inhibition	IC50: range from 2.2 to 7.9 μM for ALDH1A1, 5.4 to 8.6 μM for ALDH2, and 1.7 to 12 μM for ALDH3A1	No in vivo studies	^252^
Aldi‐6	Covalent ALDH inhibition	IC50: 600 nM for ALDH1A1, 800 nM for ALDH2, and 1 μM for ALDH3A1	The combination of Aldi‐6 and cisplatin results in a greater reduction in tumor burden in HNSCCs	^253^
Dyclonine	Covalent ALDH inhibition	IC50: 35 μM for ALDH2, and 76 μM for ALDH3A1	The combination of dyclonine and sulfasalazine efficiently suppresses the growth of ALDH3A1‐expressing HNSCC or GC tumors	^252^, ^254^

Abbreviations: DEAB, diethylaminobenzaldehyde; DIMATE, 4‐dimethylamino‐4‐methyl‐pent‐2‐ynthioic acid‐*S*‐methylester; DSF, disulfiram; GBM, glioblastoma; GC, gastric cancer; HNSCC, head‐and‐neck squamous cell carcinoma; IC50, inhibitory concentration; NSCLC, non‐small cell lung cancers; PCa, prostate cancer; TMZ, temozolomide.

DSF is an irreversible pan‐ALDH inhibitor, and it has an inhibitory concentration (IC50) of 0.15 μM for ALDH1 and an IC50 of 1.45 μM for ALDH2.^243^, ^255^ DSF is a Food and Drug Administration–approved anti‐alcoholism drug in 1951.^256^ In the past decades, both in vivo and in vitro experiments have shown that DSF has excellent treatment efficacy, and there are some clinical studies (Table 4) indicating that DSF is now used in patients with some diseases like alcohol‐related disorders and solid tumors. For example, DSF is used in clinical routine for the recrudescence prevention of alcohol dependency or severe alcoholism (NCT00431262) that acts as a “psychological deterrent” and causes unpleasant physical reactions after alcohol consumption.^257^ Some clinical trials have shown that DSF is used in combination with other drugs, such as lorazepam for the treatment of alcohol dependency and anxiety disorders (NCT00721526). Moreover, DSF treatment can increase the expression of metalloproteinase 10 (ADAM10) in peripheral blood cells of AD mice, suggesting that DSF can be repurposed as an ADAM10 enhancer and a therapeutic approach for AD.^258^ These early findings emphasize the necessity of clinical research to verify the therapeutic utility on human patients by analyzing the expression of ADAM10 in the collected blood samples (NCT03212599).

**TABLE 4:** Clinical trials for disulfiram (DSF)‐based therapy

Condition or disease	Drugs	Study phase	Status	Identifier
Alcoholism	Disulfiram	Not applicable	Unknown	NCT00431262
Alcohol‐related disorders	Disulfiram	Not applicable	Completed	NCT00142844
Cocaine‐related disorders	Naltrexone			
Alcohol dependence	Disulfiram	Not applicable	Completed	NCT00721526
Anxiety disorder	Lorazepam			
Alcohol addiction	Disulfiram	Not applicable	Completed	NCT03212599
Alzheimer's disease				
Recurrent GBM	Disulfiram/copper	Phase II	Completed	NCT03034135
	Temozolomide			
GBM	Disulfiram/copper	Phase II	Not yet recruiting	NCT01777919
	Temozolomide			
Metastatic breast cancer	Disulfiram	Phase II	Recruiting	NCT04265274
	Chemotherapy			
NSCLC	Disulfiram	Phase II/III	Completed	NCT00312819
	Chemotherapy			

Abbreviations: GBM, glioblastoma; NSCLC, non‐small cell lung cancers.

*Source*: clinicaltrials.gov website.

In solid tumors, DSF has been highlighted as a potential cancer therapy drug, and its cytotoxicity depends on copper (Cu).^259^, ^260^, ^261^ Studies have shown that DSF can form a complex with Cu (DSF/Cu), which is more readily taken up by cells and exerts cytotoxic effects on a variety of cancer cells while sparing normal cells.^244^, ^260^, ^262^, ^263^ DSF/Cu can inhibit ALDH‐positive NSCLC stem cells in vitro and tumors derived from sorted ALDH‐positive CSCs in vivo.^245^ A Phase I/II clinical trial has been completed and manifests surprisingly encouraging results that the combination of DSF with chemotherapy can prolong OS and PFS in patients with NSCLC (NCT00312819).^264^ In addition, preclinical studies have confirmed that DSF in combination with gemcitabine or programmed death‐ligand 1 (PD‐L1) antibody efficiently inhibits 4T1 breast cancer tumorigenicity and growth by targeting ALDH1A1 enriched CSCs and MDSCs, respectively.^119^ On the other hand, DSF/Cu can inhibit breast cancer metastasis by triggering apoptosis or inhibiting EMT in cultures and animal models. Based on the previous data, a Phase II clinical trial of DSF/Cu combination chemotherapy is being conducted to evaluate the therapeutic potential for patients with metastatic breast cancer (NCT04265274). A recent study highlights that ALDH1A3 is upregulated in recurrent GBM and is more resistant against temozolomide (TMZ) treatment.^151^ Mechanically, ALDH1A3 participates in detoxification of LPO‐derived reactive aldehydes after TMZ treatment. TMZ in combination with DSF/Cu rescues ALDH1A3‐mediated TMZ resistance in GBM patients in a Phase II clinical trial (NCT03034135). The treatment is well tolerated, with only 1 of 23 patients (4%) experiencing dose‐limiting toxicity.^265^ In addition to ALDH1A3, ALDH1A1 has also been shown to be a mediator of GBM resistance to TMZ and a reliable predictor of clinical outcomes. In an ongoing Phase II clinical trial, TMZ combined with DSF/Cu will be used as adjunctive and concurrent chemotherapy to treat newly diagnosed GBM (NCT01777919), but the results have not been published.

DEAB is a commonly used competitive and reversible ALDH inhibitor and is provided as a negative control compound in ALDEFLOUR assay.^95^ DEAB is a reversible substrate for ALDH1A1 and ALDH3A1 and displays competitive inhibition of ALDH1A1, ALDH1A3, ALDH1B1, and ALDH5A1. Moreover, it has a 50% IC50 of 57 nM for ALDH1A1, 1.2 μM for ALDH1A2, 3 μM for ALDH1A3, 1.2 μM for ALDH1B1, 0.16 μM for ALDH2, and 13 μM for ALDH5A1.^95^ Its role as an ALDH inhibitor has been extensively studied in breast cancer and ovarian cancer. DEAB can efficiently inhibit the growth of ovarian cancer cells and alleviate breast cancer tumor burden and pulmonary metastasis.^95^, ^248^ Besides, DEAB reduces the chemotherapeutic and radiotherapeutic resistance of ALDH^bright^CD44^high^ BCSCs and decreases the population of CD133^+^ ovarian CSCs.^266^, ^267^ However, its dependency on ALDH expression limits its use currently as an anticancer agent.^248^

DIMATE is the most potent ALDH inhibitor targeting the ALDH1 and ALDH3 subfamilies,^249^, ^268^ and it has an IC50 of 5 μM for each form.^249^ Specifically, it has an IC50 of 7 μM for the DU145 PCa cell line.^249^ In vivo, it is effective at a dose of 14 mg/kg daily given via intraperitoneal injection.^249^ Besides, DIMATE has been demonstrated to reduce tumor growth in vivo when injected intraperitoneally in melanoma models, while showing low toxicity on healthy cells.^250^

Citral, which is a natural product, shows potent inhibitory activity against ALDH1A1, ALDH1A3, and ALDH2 in breast cancer cells.^251^ Citral can block ALDH1A3‐mediated breast tumor growth via blocking its colony formation ability and gene expression regulation activity,^251^ as well as regulating apoptosis and cell‐cycle markers expression.^269^, ^270^ Moreover, nanoparticle encapsulated citral is used to specifically reduce the enhanced tumor growth of MDA‐MB‐231 cells overexpressing ALDH1A3 for the beneficial effects of encapsulation in the in vivo delivery of agents.^251^

Aldi‐1–4 is found in a high‐throughput screen for modulators of ALDH2 activity.^252^ The crystal structure of Aldi‐1–4 shows a covalent adduct with the active site cysteine of ALDH, and thus four related compounds show similar inhibition properties and time‐dependent kinetics for ALDHs.^252^ IC50 values for ALDH isozymes tested with the four compounds range from 2.2 to 7.9 μM for ALDH1A1, 5.4 to 8.6 μM for ALDH2, and 1.7 to 12 μM for ALDH3A1.^252^

Aldi‐6 is subsequently developed by Kim et al.^253^ It has an IC50 of 600 nM for ALDH1A1, 800 nM for ALDH2, and 1 μM for ALDH3A1.^253^ Treatment with Aldi‐6 results in a markedly decrease in HNSCC cell viability in vitro, and the combination of Aldi‐6 with cisplatin leads to a greater reduction in tumor burden in vivo.^253^

The oral anesthetic dyclonine is a covalent inhibitor of ALDH, and it is shown to be a weak inhibitor of ALDH2 and ALDH3A1.^254^ It has an IC50 of 35 μM for ALDH2 and 76 μM for ALDH3A1.^252^ The combination of dyclonine and sulfasalazine cooperatively suppresses the growth of HNSCC or GC tumors with high ALDH3A1 expression. However, monotherapy with dyclonine is not effective in vivo.^254^

Recently, isoform‐specific inhibitors are under investigation in cancer research, and some inhibitors can target ALDH1A1, ALDH2, and ALDH3A1. The ALDH1A1‐specific inhibitors are Cpd3, CM026, and CM037; NCT‐501, NCT‐505, and NCT‐506; 13 g and 13 h (Table 5). Cpd3, which is the indolinedione‐based analog, has an enhanced inhibitory activity for ALDH1A1, and IC50 is 20 nM, showing modest inhibition activity for ALDH2 and ALDH3A1.^271^ Cqd3 is competitive against ALDH binding, forming direct interactions with active‐site cysteine residues, particularly in ALDH1A1.^271^ However, there is no in vivo study using indolinedione‐based analogs. Morgan et al. optimized the indole group of Cpd3 to theophylline or benzothienopyrimidine groups and characterized two distinct chemical classes of inhibitors, CM026 and CM037, which shows a superior selection for ALDH1A1 inhibition.^272^ CM026 and CM037 have an IC50 of 0.8 and 4.6 μM for ALDH1A1 with no inhibition for other ALDH isoforms.^272^ These compounds can bind within the aldehyde binding pocket of ALDH1A1 and exploit the presence of a unique glycine residue to achieve their selectivity.^272^ It has been reported first that CM037 disrupts sphere formation and cell viability of ovarian cancer cells. Moreover, CM037 in combination with cisplatin moderately sensitizes ovarian cancer cells to the cytotoxic effects.^273^ Furthermore, CM037 could sensitize SKOV‐3‐TR, a paclitaxel‐resistant ovarian cancer cell line, to paclitaxel treatment when they are used in combination, whereas monotherapy with either agent is ineffective.^274^ Yang et al. discovered a new series of theophylline‐based analogs as potent ALDH1A1 inhibitors, and NCT‐501 is one of them.^275^ NCT‐501 has an IC50 of 0.04 μM for ALDH1A1 with no inhibition of other ALDH isoforms.^275^ Administration of NCT‐501 can reduce sphere formation and migratory potential of HNSCC cells, which is also cytotoxic against cisplatin‐resistant HNSCC cancer cells.^276^ NCT‐501 also sensitizes SKOV‐3‐TR cells to paclitaxel treatment when they are used in combination.^274^ However, NCT‐501 has limited oral bioavailability owing to metabolism by the liver before entering the systemic circulation, which limits its application in oral treatment.^275^ Yang et al. also devised a series of quinoline‐based ALDH1A1 selective inhibitors, and NCT‐505 and NCT‐506 are two such compounds.^274^ NCT‐505 and NCT‐506 both have an IC50 of 7 nM for ALDH1A1, showing high selectivity over other ALDH isozymes.^274^ NCT‐505 and NCT‐506 also sensitize SKOV‐3‐TR cells to paclitaxel treatment when they are used in combination,^274^ and subsequent studies show that NCT‐505 and NCT‐506 have reasonable systemic drug exposure when administered orally.^274^ Huddle et al. explored the structural determinants of ALDH1A isoform selectivity in a series of small molecule inhibitors, and 13 g and 13 h are discovered.^277^ An amount of 13 g has an IC50 of 80 nM for ALDH1A1, 250 nM for ALDH1A2, and 120 nM for ALDH1A3, whereas 13 h has an IC50 of 270 nM for ALDH1A1, 480 nM for ALDH1A2, and 130 nM for ALDH1A3.^277^ A period of 13 h shows synergy with cisplatin in patient‐derived ovarian cancer spheroids, and both 13 g and 13 h deplete the CD133^+^ putative stem cells in a dose‐dependent manner.^277^ However, the efficacy of these compounds in vivo has not yet been reported.^277^

**TABLE 5:** Isoform‐specific ALDH inhibitors

Compound name	Mechanism of action	Cellular activity	Animal studies	Ref.
Cpd 3	Competitive ALDH1A1 inhibition	IC50: 20 nM for ALDH1A1	No in vivo studies	^271^
CM026	Competitive ALDH1A1 inhibition	IC50: 0.8 μM for ALDH1A1	No in vivo studies	^272^
CM037	Competitive ALDH1A1 inhibition	IC50: 4.6 μM for ALDH1A1	Ineffective in vivo	^272^, ^273^, ^274^
NCT‐501	Competitive ALDH1A1 inhibition	IC50: 0.04 μM	Ineffective in vivo	^274^, ^275^, ^276^
NCT‐505/NCT‐506	ALDH1A1 inhibition	IC50: 7 nM for ALDH1A1	NCT‐505 and NCT‐506 have reasonable systemic drug exposure when administered orally	^274^
13 g/13 h	Noncovalent, noncompetitive ALDH1A inhibition	IC50 (13 g): 80 nM for ALDH1A1, 250 nM for ALDH1A2, 120 nM for ALDH1A3; IC50 (13 h): 270 nM for ALDH1A1, 480 nM for ALDH1A2, 130 nM for ALDH1A3	No in vivo studies	^277^
CVT‐10216	Reversible ALDH2 inhibition	IC50: 29 nM for ALDH2	No in vivo studies	^278^
ALDH423	ALDH2 inhibition	IC50: 0.62 μM for ALDH2	No in vivo studies	^279^
CB7	Competitive ALDH3A1 inhibition	IC50: 0.2 μM for ALDH3A1	No in vivo studies	^280^
CB29	Reversible ALDH3A1 inhibition	IC50: 16 μM for ALDH3A1	No in vivo studies	^281^
Acivicin	Covalent ALDH4A1 inhibition	IC50: 5.4 μM for ALDH4A1	No in vivo studies	^282^

Abbreviation: IC50, inhibitory concentration.

The ALDH2‐specific inhibitors are CVT‐10216 and ALDH423. CVT‐10216 is a highly selective, reversible inhibitor of ALDH‐2, and it has an IC50 of 29 nM for ALDH2.^278^ A target‐specific rescoring method is applied for the search of small molecule inhibitors of the mitochondrial ALDH2, and ALDH423 has found an IC50 of 0.62 μM for ALDH2.^279^ As ALDH2 is not found to be crucial during carcinogenesis, CVT‐10216 and ALDH423 have not yet been tested in cancer models.^178^

The ALDH3A1‐specific inhibitors are CB7 and CB29. CB7 is identified by Parajuli et al. in a high‐throughput screen. Enzyme kinetics and crystallographic studies show that CB7 is competitive with respect to aldehyde binding and noncompetitive with respect to NADP^+^ binding of ALDH3A1, and it has an IC50 of 0.2 μM for ALDH3A1.^280^ Treatments of the ALDH3A1‐expressing LUAD cell A549 and GBM cell SF767 with mafosfamide in combination with 10 μM CB7 enhance the antiproliferative effects over monotherapy.^280^ CB29 is a reversible ALDH3A1 inhibitor, which has an IC50 of 16 μM for ALDH3A1.^281^ And it also binds within the aldehyde substrate‐binding site of ALDH3A1 like CB7.^281^ The therapeutic effect of CB29 is similar to that of CB7 when in combination with mafosfamide.^281^ However, no in vivo study has been reported with both CB7 and CB29.

There are other ALDH isoform inhibitors like Acivicin, which is a natural product and inhibit ALDH4A1 activity by binding to the catalytic site, and it has an IC50 of 5.4 μM for ALDH4A1.^282^ Cytotoxicity of Acivicin is investigated in HCC cells, which shows an IC50 for ALDH4A1 of 0.7 μM,^282^ while in vivo study has not been reported nowadays.

### 5.2 Targeting ALDHs via inhibiting ALDH‐related molecular pathways

**FIGURE 2:**
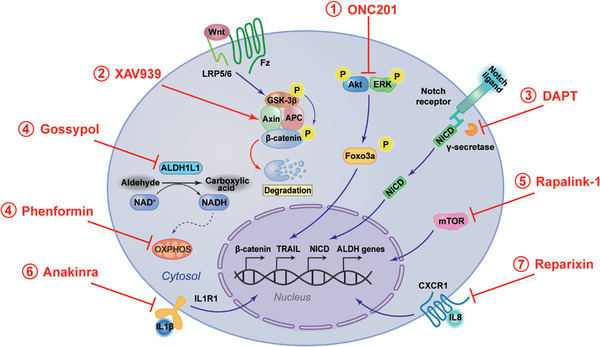
Therapies targeting aldehyde dehydrogenases (ALDHs)‐related molecular pathways. (1) ONC201 dually inhibits phosphorylation of Akt and extracellular regulated–protein kinase (ERK), leading to the dephosphorylation of transcription factor Foxo3a. Dephosphorylated Foxo3a translocates into the nucleus where it activates transcription of its target genes, including pro‐apoptotic death receptor ligand TNF‐related apoptosis‐inducing ligand (TRAIL), ALDH1A1, and ALDH7A1. (2) WNT signaling inhibitor, XAV939 stimulates β‐catenin destruction by stabilizing Axin, which finally leading to significant inhibition of the ALDH‐positive populations. (3) Pharmacological inhibition of Notch pathway by using a γ‐secretase inhibitor, DAPT reduces ALDH‐positive tumor cells. (4) Dual inhibition of ALDH1L1 and oxidative phosphorylation (OXPHOS) by gossypol and phenformin reduces tumor bioenergetics and stemness. (5) Rapalink‐1 can block mTORC1/2 signaling and reduce ALDH‐positive populations. (6) IL‐1 receptor (IL1R1) antagonist, anakinra can decrease ALDH and IL1R1 double positive cancer stem cells (CSCs). (7) Reparixin can reduce the metastatic behavior of cancer cells and eradicate the ALDH‐positive populations by inhibiting IL8‐CXCR1 signaling

Targeting the ALDH‐related molecular pathways is another promising strategy to inhibit tumor progression and CSC self‐renewal. Previous studies demonstrate that multiple molecular pathways such as RA, Notch, Wnt, and TGF‐β pathways may regulate ALDHs activity (Figure 2). A good example is that Wnt/β‐catenin signaling activates ALDH1A1 transcription, and this signaling could also regulate the maintenance of CSCs and drives radio‐therapeutic resistance in PCa patients. Blocking Wnt/β‐catenin signaling by XAV939 antagonist leads to significant inhibition of the ALDH‐positive populations and re‐sensitization of the PCa cells to radiation therapy.^283^ Notably, ONC201, which is a well‐tolerated compound used in a Phase I/II study for patients with advanced solid tumor (NCT02038699) (NCT02324621),^284^ has been reported to downregulate CSC‐related genes, including ALDH1A1 and ALDH7A1, and suppress CSCs self‐renewal in CRC, PCa, and GBM cells.^285^ Rapalink‐1, a third‐generation mTOR inhibitor, is found to block mTORC1/2 signaling,^286^ accompanied by the reduction of the proportion of ALDH‐positive cells in PCa patient‐derived organoids and decreased tumor burden in xenograft mouse models.^287^ CXCR1, one of the receptors for CXCL8 (IL‐8), is identified in ALDH1‐positive breast cancer CSCs. Administration inhibitor of CXCR1, reparixin, can reduce the metastatic behavior of cancer cells and eradicate the CSC populations both as a single agent and in combination with chemotherapy.^288^ However, in a randomized, double‐blind, placebo‐controlled Phase II clinical trial (NCT02370238), the combination of reparixin and paclitaxel does not improve PFS of TNBC patients over paclitaxel alone, which may warrant sufficient samples in the future. Notch signaling is important for the maintenance of ALDH1A1 positive CSCs, and pharmacological inhibition of Notch pathway by using a γ‐secretase inhibitor, DAPT reduces ALDH^+^ tumor cells^197^, ^289^ in lung and breast cancer. Interestingly, ALDH and IL‐1 receptor (IL1R1) double‐positive CSCs are enriched following antiestrogen therapy and held responsible for the treatment failure in breast cancer patients, which can be attenuated with IL1R1 antagonists such as anakinra.^290^ Combinatorial therapy with gossypol and phenformin, which target ALDH1L1 and oxidative phosphorylation, respectively, results in ATP depletion in GBM spheres. In addition, this dual inhibition of tumor bioenergetics markedly attenuates GBM cell stemness and invasion ability in a preclinical mouse model.^285^ Noteworthy, decreased ALDH1A2 expression is associated with the acquisition of invasive capacity and stem‐cell traits due to impaired ALDH1A2‐RAR‐dependent signaling. Administration with retinoids inhibits malignancy progression in HNSCC patient‐derived xenografts.^129^ Low expression of ALDH1A2 in cancers is always due to its promoter hypermethylation.^128^, ^132^

### 5.3 Targeting ALDHs may promote immunotherapeutic efficiency

Immunotherapy has recently drawn global attention as the “new hope” for cancer treatment.^291^ Several studies provided evidence that some ALDH isoforms might contribute to tumor immune surveillance.^292^ Targeting of ALDH‐positive CSCs through immunological approaches is also currently underway in the treatment of cancer. In particular, ALDH3A1 enzyme‐enriched CSCs populations are positively correlated with PD‐L1 expression in melanoma and NSCLC.^190^ GC mesenchymal stem cells maintain a pool of CSCs through increased the expression level of PD‐L1, which leads to the chemotherapeutic resistance of GC cells. Blocking PD‐L1 expression by neutralizing antibody in GC cells inhibits their sphere formation ability and ALDH activity.^293^, ^294^ More recently, Liu et al. revealed that ALDH1A1 results MDSCs infiltration in 4T1 breast tumor mouse models, and the administration of DSF robustly enhances the therapeutic efficiency of PD‐L1 monoclonal antibody in vivo. Zhang et al. found that ALDH2 mediates the immune evasion induced by alcohol in CRC by stabilizing PD‐L1 protein.^119^ It is also noteworthy that the combination of ALDH2 inhibition and anti‐PD‐1 antibody enhances antitumor immunity and tumor eradication, which may serve as a novel strategy to enhance the efficacy of immune checkpoint blockade in CRC patients, especially in those who consumed alcohol.^295^ The development of anti‐ALDH vaccines can be an efficient strategy to eradicate CSC populations and improve the therapy response.^296^ Hassani Najafabadi et al. used a nanoparticle‐based vaccine to deliver ALDH1A1 and ALDH1A3 dual peptides to antigen‐presenting cells and generated robust ALDH‐specific T‐cell responses against ALDH^bright^ CSCs. Combined with anti‐PD‐L1 therapy, the anti‐ALDH vaccination exerts potent antitumor efficacy in murine models of D5 melanoma and 4T1 breast cancer.^297^ Similarly, ALDH dual peptides‐DC vaccination plus anti‐PD‐L1 administration result in an increased recruitment of CD3^+^ tumor‐infiltrating lymphocytes in the residual melanoma tumors and a further reduction of ALDH^bright^ CSCs. Thus, targeting CSCs with these vaccines in combination with ICIs may be the new avenues for preventing tumor relapse and increasing patient survival.^298^

## 6 OUTLOOK

Throughout the past decades, pioneering studies support that aberrant ALDH activity or expression is distinctly associated with neurological abnormalities, metabolic diseases, and especially in solid tumors. For example, ALDHs promote the oxidation of anticancer drugs, such as cyclophosphamide, into less toxic metabolites, which causes chemotherapeutic resistance. ALDHs participate in RA synthesis associated with cancer cell proliferation and immune system regulation. Besides, ALDHs play key roles in CSC‐mediated tumor recurrence and metastatic dissemination, indicating poor clinical outcomes in patients. However, the molecular mechanisms underpinning aberrant ALDH activity or expression during malignant transformation and progression are fragmented and limited, which warrants further investigation. The exploration of ALDHs more detailed functions and regulation mechanisms will also promote our understanding of other diseases like PD and AD.

Accumulating evidence indicates that ALDHs can be successfully considered diagnostic markers for certain diseases. Preclinical researches demonstrate that ALDH1A1 is an optimal predictor for PD risk. Besides, the clinical assessment of ALDH2 as a marker will be beneficial to the diagnosis of cardiac anomalies. In addition, ALDHs are considered promising biomarkers of CSCs with functional and mechanistic involvement in tumor initiation and progression as well as in modulating their response to tumor therapies.^299^ Thus, progress in identifying and quantifying ALDHs as risk factors or markers is opening the way to the prevention of disease and maintenance of health.

The existence of CSCs in majority of solid tumors has important clinical implications for the development of inhibitors and design of clinical trials to assess treatment efficacy. Therefore, an ideal ALDH‐targeting inhibitor should be nontoxic and well tolerated that can be safely administered by patients and in combination with conventional therapies, such as chemotherapy, radiotherapy, and immunotherapy to improve disease control over non‐CSC, bulk tumor cells. Isoform‐specific ALDH inhibition combats the potential problem of toxicity of multi‐ALDH isoform inhibition due to the wide distribution of ALDH enzymes in normal tissues. However, this will require further in vivo testing as monotherapy due to the compensatory activity by other isoforms. Nevertheless, the multi‐ALDH inhibitors approach remains the most promising for translation into the clinic because more than one ALDH isozyme expresses in CSCs. Several strategies in ALDH inhibitor delivery systems are now employed in multiple cancer types to reduce off‐target toxicities in normal cells.

The findings of several studies indicate that CSCs with high ALDH activity harbor important alterations affecting oncogenic pathways including the Notch and Wnt pathway, mediating the resistance of cancers to therapy, tumor recurrence, and metastasis.^300^ In the context of clinical oncology, several clinical trials using single‐agent and/or combination therapies have been performed to study the safety and efficacy of targeting ALDH‐related molecular pathways.^301^ In addition, multiple observations highlight the interactions between ALDH‐positive CSCs and the immune system, which contributes to immunosuppression and stemness phenotype. Therefore, urgent preclinical or clinical studies may require a combination of ALDH‐specific and immune‐based therapies in the future.^302^ Finally, due to the availability of large amounts of clinical information and primary clinical data, it is critical to consider repurposing “old” drugs to treat both common and rare diseases.^303^ For example, the anti‐alcoholism drug DSF has attracted increasing attention for its anticancer effects. In the long term, patients will benefit from drug repurposing in translational medicine.

## AUTHOR CONTRIBUTIONS

Jie Xia wrote the first draft of the manuscript. Siqin Li contributed to drawing figures. Lixing Zhang and Suling Liu contributed to manuscript revision. All authors contributed to the article and approved the submitted version.

## CONFLICT OF INTEREST

The authors declare no conflict of interest.

## ETHICS STATEMENT

Not applicable.

## Data Availability

Not applicable.
